# Supplementary material for: E2F1–3 activate Merkel cell polyomavirus early transcription and replication
Source: bioRxiv. 2025 Dec 17:2025.11.15.688648. Originally published 2025 Nov 16. Preprint. [Version 2] doi: 10.1101/2025.11.15.688648 (PMC12642449; doi:10.1101/2025.11.15.688648)
Supplement: Supplement 2 [file media-2.pdf]

Supplementary table 1. sgRNA sequences.

**Primer name**	**Sequence (5' -> 3')**
sgNTC	GTGTAGTTCGACCATTCGTG
sgF1	GTATCTAAGGGCAGATCCCA
sgF2	ATAAAAACCACTCCTTAGTG
sgR1	ATACTGCAGTTTCCCGCCCT
sgR2	GCAAATGAGCTACCTCACTA

Supplementary table 2. Sequences of 5'-biotinylated primers used to generate DNA pulldown probes

dsDNA probe	F primer (5' -> 3')	R primer (5' -> 3')
AmpR	TAGTGCTGCCATTACCATGAGC	TTACCAGTGCTTGATCAGTGAGG
MCPyV NCCR	CCCCATCCTGAAAAATAAATAAG	GACTAAATCCATCTTGTCTATATGC
Gorilla PyV NCCR	CCTGAAAAATAAATAAGGATTACTT	GTTGGTGGAGCTCTGCAAGCAAAATG
Otomops PyV NCCR	TCTGAAAAAAAAAAAACATGTACTC	GGCTGAAGAGGCTCTAGAGATCCTG
Murine PyV (A2) NCCR	TTTGAAAATTCACTTACTTGATCAG	GATGGTGGTGAGGCTGAAATGAGGC
Hamster PyV NCCR	AGTTATTAATGAAGTAACTTGGGC	CTTGCTTGTTGCAGCTAGAGATGC
TSPyV NCCR	TTTACCTGAAAAATAAGAAAAACTTACC	TTTTGCTGAATGCACCAGAAGACAGG
BKPyV (NEB-10) NCCR	GCCTTTGTCCAGTATTAACTGGGG	TTTTGCAAAAAATTGCAAAAGATTAGG
JCPyV (CY) NCCR	GGCCAGCTGGTGACAAGCCAAAACAGC	TTTAGCTTTTTGCAGCAAAAAATTAG
SV40 NCCR	GGCCTGAAATAACCTCTGAAAG	CTTTGCAAAGCTTTTTGCAAAAGCCTAGG

Supplementary table 3. NCCR sequences for pulldown experiments and luciferase vectors. E2 sites are highlighted and underlined.

**dsDNA probe**	**NCBI GenBank ID:**	**Sequence (5' -> 3')**
AmpR	-	TAGTGCTGCCATTACCATGAGCGACAACACCGCGGCCAACT<br>TACTTCTGACAACGATCGGAGGCCCTAAGGAGCTGACTGCA<br>TTTCTTCATAATATGGGTGATCATGTGACCCGGCTTGACCGC<br>TGGGAACCAGAGTTGAACGAAGCCATACCGAACGACGAGC<br>GTGATACCACGATGCCAGTAGCAATGGCCACAACTCTTCGG<br>AAACTACTCACTGGCGAACTTCTTACTCTAGCATCACGACAG<br>CAGCTCATAGACTGGATGGAGGCGGACAAAGTAGCAGGACC<br>ACTTCTTCGCTCGGCCCTCCCTGCTGGCTGGTTCATTGCTG<br>ACAAATCGGGGGCCGGTGAACGCGGCTCTCGCGGCATCATT<br>GCTGCGCTGGGGCCTGATGGTAAGCCCTCACGAATCGTAGT<br>GATCTACACGACGGGGAGTCAGGCCACTATGGACGAACGAA<br>ATAGGCAGATCGCTGAGATCGGTGCCTCACTGATCAAGCACT<br>GGTAA
MCPyV NCCR	HM011549.1	CCTGAAAAATAAATAAGGATACTTACTCTTTTAATGTCCTCCTC<br>CCTTTGTAAGAGAAAAAAAAGCCTCCGGGCCTCCCTTGTTG<br>AAAAAAAGTTAAGAGTCTTCCGTCTCCCTCCCAAACAGAAAG<br>AAAAAAAGTTTTGTTTATCAGTCAAACTCCGCCTCTCCAGGA<br>AATGAGTCAATGCCAGAAACCCTGCAGCAATAAAAGTTCAAT<br>CATGTAACCACAACTTGGCTGCCTAGGTGACTTTTTTTTTTCA<br>AGTTGGCAGAGGCTTGGGGCTCCTAGCCTCCGAGGCCTCT<br>GGAAAAAAAAGAGAGAGGCCTCTGAGGCTTAAGAGGCTTAA<br>TTAGCAAAAAAGGCAGTATCTAAGGGCAGATCCCAAGGGCG<br>GGAAACTGCAGTATAAAAACCACTCCTTAGTGAGGTAGCTCA<br>TTTGCTCCTCTGCTCTTTCTGCAAACTCCTTCTGCATATAGAC<br>AAG
Gorilla PyV NCCR	NC_025380.1	CCTGAAAAATAAATAAGGATTACTTACTCAGCCTTGTCCTCCT<br>CCCTTTGTAAGAGAAAAAAAAGGAGTCTTCTCGCTTCCCTCC<br>TCCCTTTTGAAGAAAAAAAAATGCTGCGTCGCTCTCCCCGCT<br>TGTCGCCTCCCTTTGTGTTGAAAAAAAGTTGTGTTAAGAGTC<br>TACTTCCTCCCTCCCAACTAGATTTAAAAAAATTGTTTATTATAT<br>AACTCCGCCTCTCCAGGATATGAGTCAATGCCAAGAAGCCT<br>GCAGCAATAAAAGTTCAATCAGAGTAAAACCACAAGCTGTCT<br>GCCAGACCACAAGCGTTGCCTAGGCAGCCTATTTTTTTTTAC<br>AAATTAGTGCGAGGCTTGGGGCTCCTAGCCTCCGAGGCCTC<br>TGGAAAAAAATAGTGAGAGGCCTCTGAGGCCTCTAACAGCTTA<br>ATTAGCAGAACCATTCCTGGGCGGGAAACTGCAGTATAAAAG<br>CCACTCCTAAGTGATGTAGCTCATTTTGCTTGCAGAGCTCCA<br>CCAAC
Otomops PyV NCCR	NC_020071	TCTGAAAAAAAAAAAACATGTACTCACTTTTAATGCCTCCGCC<br>CGTTCAGAAAGAAAAAAATCCACTCGGCGCTGGGGCTCCCG<br>CCCGCTCTGTTTAAAAAAAATGTTTGAAATGGTTGCTGACCT<br>CCTCCCTTCGTGCTTAGAAAAAATCCTACTCATCATGACTAAC<br>CCCGCCCGCAGAGACAGAAAAAAACAATTTAAAAGGCTGCA<br>GTAAGGAAATGACTCATTCGTGCCGGCGCCTGAACCAAATG<br>ACAGGGGGAGCTCTTTTTTTTTTCAAGTATGCAGAGGCTAGA<br>GGCCCTTAGCCCCTGAGGCTTTCACAGAAAAAGTAGAGAGG<br>CCCTGGGAGGCTTTTTTTAAATTATAGCCGTTAATAGGCGGG<br>AAGGGCTGGTATAAAAGCCTGTTATTCTCCTCCTCAGGATCT<br>CTAGAGCCTCTTCAGCC

dsDNA probe	NCBI GenBank ID:	Sequence (5’ -> 3’)
Murine PyV (A2) NCCR	J02288.1	TTTGAAAATTCACTTACTTGATCAGCTTCAGAAGATGGCGGA<br>GGGCCTCCAACACAGTAATTTTCCTCCCGACTCTTAAAATAG<br>AAAATGTCAAGTCAGTTAAGCAGGAAGTGACTAACTGACCGC<br>AGCTGGCCGTGCGACATCCTCTTTTAATTAGTTGCTAGGCAA<br>CTGCCCTCCAGAGGGCAGTGTGGTTTTGCAAGAGGAAGCAA<br>AAAGCCTCTCCACCCAGGCCTAGAATGTTTCCACCCAATCAT<br>TACTATGACAACAGCTGTTTTTTTTAGTATTAAGCAGAGGCCG<br>GGGGCCCCTGGCCTCCGCTTACTCTGGAGAAAAAGAAGAG<br>AGGCATTGTAGAGGCTTCCAGAGGCAACTTGTCAAAACAGG<br>ACTGGCGCCTTGGAGGCGCTGTGGGGCCACCCAAATTGATA<br>TAATTAAGCCCCAACCGCCTCTTCCCGCCTCATTTCAGCCTC<br>ACCACCATC
Hamster PyV NCCR	NC_001663.2	AGTTATTAATGAAGTAACTTGGGCATCTATCGCGGCAAAGGC<br>TTCTCCACTAAGTATGGCCTCTACTGAAATTCCAGTAACTGAT<br>GAAATTTCGGAGAGGTAGCTGATCATCTCAATAATCACTGAAA<br>TGGCAGATCCCATGTTGACTTACTTGAACAGTTTGAAAATCTT<br>CTGAACTGTTTCAGGCAGGTTTTTAGGCCGAATTCTAAAGAA<br>ACAGAAAGCAAACACTCAGCGCCGAAGAGCAGGAAATGGCT<br>GACCACTGCACTTGGGCGACACGACACGCCTAGCGATAAGG<br>AAGTCACCATGGCAACATAACCGCAGCACTGCTGTTGTCACA<br>GTTGCCTAGCAAATGACAGACTCAGCAACCACAGGAGAGGA<br>AATGATAGGGCTAGCATTTTTTCAAATGTAAACCAGAGGCTAG<br>GGGCCCCTTGCCTCCTTAGCTCTCAAGTAGAAAAGGAAGAG<br>AGGCTTTTGGGGCTTTTTGGCTTTAAGCCTCATTTTATGAGC<br>AGGAGGAGCTTGTTGCAACTTGAGAGGCGTTTTGAGGCTTC<br>CAGGCAGAGAATACTCACAGACCCCACACAGTCTAGACGCT<br>CAGAAGCATCTCTAGCTGCAACAAGCAAG
TSPyV NCCR	NC_014361.1	TTTACCTGAAAAATAAGAAAAACTTACCATTAGAATTTGAATTT<br>GCCGCGTGAGAACACAGAGCGGGAGGATGTGTTGTTATGGA<br>GACCGGACGGACAGGATGAGAGCAGTTAGACAAGTGCTATC<br>TTAAGAATATACAAAAACAATCACGGGATTTGGCTGGCTTCCT<br>CTTGTCTCTTGGCTGCCTTCCTGTGTTTGTGGCAGTATGACA<br>AACATCCCCTTGAGACTAACTGACACAGCATTTTCCATAACAA<br>ATGATAGGTGGCGCACGAGCTTCCTCAGGATAACGGTCTTAA<br>GCAAACAATGACCTATTTATGTCAACACTCTAAGTATAGTTCC<br>TCAAATTCCTAATAGGGGGTTACTATGGAAATCTTGTTTTTTCT<br>CAGTATTACCAGGAGGCTGAGGCTTCCTGCCCCCTCTTGAC<br>ACAGACATAGAGGGAGAGGCCTCCGGAGGCCTCTTGAGGC<br>TTGGCACTTCCTCCCTTTTATGTAAGCCAAATCTGATGAAAAT<br>TAATTGAGGCACACAGAGGCTTTAAAAGGGCTCCAAAAAGCT<br>CCCATCTTCATTTCTTCATTTCTCCCCTTACCTGTCTTCTGGT<br>GCATTCAGCAAA
BKPyV (NEB-10) NCCR	AB365141.1	GCCTTTGTCCAGTATTAACTGGGGACAAGGCCAAGATTCCTA<br>GGCTCGCAAAACATGTCTGTCTGGCTGCTTTCCACTCCTTTG<br>GCTAGTTTCCACTTCCTCTTGTGTTTATTTGAGAATTCTAGGG<br>GCGGGGTTTCACTATTAACTGCCACTGGCTGGCTGCCCAGT<br>CATGCACTTTCCTTCCTGAGGTCATGGTTTGGCTGCATTCCA<br>TGGGAAAGCAGCTCCTCCCTGTGGCCTTTTTTTTTATAATATA<br>TAAGAGGCCGAGGCCGCCTCTGCCTCCACCCTTTCTCTCAA<br>GTAGTAAGGGTGTGGAGGCTTTTTCTGAGGCCTAGCAAAAC<br>TATTTGGGGAAATCCCTAATCTTTTGCAATTTTTTGCAAAA

dsDNA probe	NCBI GenBank ID:	Sequence (5’ -> 3’)
JCPyV (CY) NCCR	AB038249.1	GGCCAGCTGGTGACAAGCCAAAACAGCTCTGGCTCGCAAAA<br>CATGTTCCCCTGGCTGCTTTCCACTTCCCCTTGTGCTTTGTT<br>TACTTGTGATTAAGGACTATGGGAGGGGTTTCACTATAACTGC<br>CAGTGGCATGCAGCCAGGGCTCCCTCTGGCTGTCAGCTGG<br>TTGGCTCCCTAGGTATGAGCTCATGCTTGGCTGGCAGCCATC<br>CAGTTTTAGCCAGCTCCTCCCTACCTTCCCTTTTTTTTATATAT<br>ACAGGAGGCCGAGGCCGCCTCCGCCTCCAAGCTTACTCAG<br>AAGTAGTAAGGGCGTGGAGGCTTTTTAGGAGGCCAGGGAAA<br>TTCCCTTGTTTTTCCCTTTTTTGCACTAATTTTTTGCTGCAAAA<br>AGCTAAA
SV40 NCCR	J02400.1	GGCCTGAAATAACCTCTGAAAGAGGAACTTGGTTAGGTACCT<br>TCTGAGGCGGAAAGAACCAGCTGTGGAATGTGTGTCAGTTA<br>GGGTGTGGAAAGTCCCCAGGCTCCCCAGCAGGCAGAAGTA<br>TGCAAAGCATGCATCTCAATTAGTCAGCAACCAGGTGTGGAA<br>AGTCCCCAGGCTCCCCAGCAGGCAGAAGTATGCAAAGCATG<br>CATCTCAATTAGTCAGCAACCATAGTCCCGCCCCTAACTCCG<br>CCCATCCCGCCCCTAACTCCGCCCAGTTCCGCCCATTCTCC<br>GCCCCATGGCTGACTAATTTTTTTTATTTATGCAGAGGCCGAG<br>GCCGCCTCGGCCTCTGAGCTATTCCAGAAGTAGTGAGGAGG<br>CTTTTTTGGAGGCCTAGGCTTTTGCAAAAAGCTTTGCAAAG

Supplementary table 4. qPCR primers.

**Primer name**	**Sequence (5’ -> 3’)**
PanT-7 F	ATGGCAACATCCCTCTGATGA
PanT-7 R	TGGAATTTGCTCCAAAGGGTG
36B4 F	TGCCAGTGTCTGTCTGCAGA
36B4 R	ACAAAGGCAGATGGATCAGC
VP1 F	AAAACACCCAAAAGGCAATG
VP1 R	GCAGAGACACTCTTGCCACA
GAPDH F	TGTGTCCCTCAATATGGTCCTGTC
GAPDH R	ATGGTGGTGAAGACGCCAGT

Supplementary dataset 1. MKL-2 DNA pulldown mass spectrometry dataset. Fold change (FC) and p values for proteins detected by mass spectrometry following AmpR and NCCR pulldowns. $\log_2$(FC), and $-\log_{10}$(p) are plotted in Figure 1B.

Supplementary dataset 2. 293A DNA pulldown mass spectrometry dataset. Fold change (FC) and p values for proteins detected by mass spectrometry following AmpR and NCCR pulldowns. $\log_2$(FC), and $-\log_{10}$(p) are plotted in Figure 2A.
